# Vulnerable Workers and COVID-19: Insights from a Survey of Members of the International Commission for Occupational Health

**DOI:** 10.3390/ijerph18010346

**Published:** 2021-01-05

**Authors:** Jacques Tamin, Oluranti Samuel, Anna Suraya, Ikenna D. Ebuenyi, Nisha Naicker, Minha Rajput-Ray

**Affiliations:** 1 Centre for Occupational and Environmental Health, University of Manchester, Oxford Rd, Manchester M13 9PL, UK; 2 Department of Sociology, Faculty of Social Sciences, Lagos State University, Ojo, Lagos State 10001, Nigeria; oluranti.samuel@lasu.edu.ng; 3 Faculty of Public Health, Binawan University, East Jakarta, Jakarta 13630, Indonesia; anna_suraya2005@yahoo.com; 4 Assisting Living & Learning (ALL) Institute, Department of Psychology, Maynooth University, Maynooth, Ireland; ikenna.ebuenyi@mu.ie; 5 National Institute for Occupational Health, National Health Laboratory Services, Johannesburg 2000, South Africa; nishan@nioh.ac.za; 6 NNEdPro, Global Centre for Nutrition and Health, Global Centre for Nutrition and Health, St John’s Innovation Centre, Cambridge CB4 0WS, UK; m.rajput-ray@nnedpro.org.uk

**Keywords:** COVID-19 pandemic, disadvantaged populations, vulnerable populations, workers, social justice, social determination of health, poverty, public health practice

## Abstract

The COVID-19 pandemic has negatively impacted on the health and wellbeing of populations directly through infection, as well as through serious societal and economic consequences such as unemployment and underemployment. The consequences could be even more severe for those more vulnerable to the disease, such as the elderly and those with underlying health conditions. Indeed, there is evidence that such vulnerable populations are disproportionately affected in terms of both, their health and the socioeconomic impact. The aim of our study was to determine whether occupational health (OH) professionals thought that the COVID-19 pandemic might further disadvantage any particular group(s) of vulnerable workers globally, and if so, which group(s). A cross-sectional study was carried out with a sample of OH professionals by means of an online questionnaire which was shared via email within the ICOH (International Commission for Occupational Health) community. Data was collected over a period of two weeks in May 2020 and 165 responses from 52 countries were received. In this paper, the responses relating to questions about vulnerable workers are reported and discussed. Globally, our responders felt that those in less secure jobs (precarious employment (79%) and informal work (69%)), or unemployed (63%), were the most at risk of further disadvantage from this pandemic. The majority felt that their governments could act to mitigate these effects. There were suggestions of short-term alleviation such as financial and social support, as well as calls for fundamental reviews of the underlying inequalities that leave populations so vulnerable to a crisis such as COVID-19.

## 1. Introduction

The COVID-19 pandemic has affected and continues to affect the lives of millions globally [1]. It has negatively impacted on the health and wellbeing of populations directly through infection, as well as through serious societal and economic consequences such as unemployment and underemployment [2]. Furthermore, there is evidence that vulnerable populations are disproportionately affected in terms of both, their health and the socioeconomic impact [2,3]. Individuals with increased vulnerabilities to the disease include people with disabilities, the elderly, and people living in poverty [3]. Groups at greater risk of suffering from economic loss include those with underlying health conditions, older people, women, young persons, as well as migrant and unprotected (e.g., casual) workers [2]. Even before this pandemic, “vulnerable workers” were recognized as those in higher risk occupations or vulnerable because of their health or socioeconomic circumstances [4]. Therefore, someone with an underlying health condition would be more vulnerable to more serious health detriments from COVID-19, as well as being at greater risk of poverty as a result of this pandemic. Moreover, disadvantaged individuals may have lower educational attainment, have poorer health, and live at or below the poverty line [5]. Thus, such disadvantages can compound one another. It has been argued that this compounding effect increases the likelihood of such individuals becoming vulnerable workers (for example, they may be in more insecure work) [6].

To illustrate this, let us consider in greater detail one of these groups, namely those individuals with long term health conditions or disabilities. For example, if they had a severe respiratory condition such as chronic obstructive pulmonary disease (COPD), they could be more susceptible to serious COVID-19 complications [7]. Such individuals, along with those suffering from many other health conditions and disabilities, could be more severely impacted by this pandemic in terms of their health and wellbeing. Furthermore, they might have to take greater preventive measures to avoid being infected (such as socially isolating themselves, especially as some workplaces may pose an increased COVID infection risk [8]), and thus could suffer more in socioeconomic terms. Even before the pandemic, it was noted that those with disabilities and long-term health conditions were significantly disadvantaged in the workplace, sometimes facing discrimination and receiving lower rates of pay [9]. Disability is also associated with lower educational attainment, higher morbidity, lower employment rates, and poverty [10]. The disabled suffer further handicap in that they often need higher earnings to achieve the same standard of living as an able-bodied person [11,12], for example, due to their higher heating, care, and transportation costs. Therefore, if the COVID pandemic is affecting the socioeconomic wellbeing of populations globally, is it possible that those who were already disadvantaged, such as the disabled, would suffer even more than others (that is, populations not considered to be more vulnerable)?

In our opinion, occupational health (OH) professionals could provide valuable insights to this question. Occupational health is the health discipline that focuses on the interface between work and health, particularly concerning the effects of work on health and the health and fitness of individuals to work in specific jobs. Occupational health professionals include physicians and nurses, as well as others such as physiotherapists, ergonomists, and occupational hygienists [13]. Therefore, professionals working in this field will have intimate experience of the impact of work (or lack of work) on the health of workers, including those in disadvantaged groups, in circumstances such as this pandemic. This may be the case whether the OH professional is a clinician, academic, researcher, or works at policy level, as they are all interested in this interplay between work and health. The primary aim of OH is to protect and promote workers’ health [14]. The International Commission for Occupational Health (ICOH) is the oldest global scientific association in this field and has over two thousand members in 93 countries [15]. We believe that the views of ICOH members would, hence, be particularly appropriate for evaluating the effects of this pandemic on vulnerable workers in the context of health and work (including unemployment and underemployment).

The aim of this study was to determine whether our respondents perceived that any groups of vulnerable workers would suffer greater detriment as a result of the pandemic, and if so, which groups.

## 2. Methods

The study was undertaken by members of the ICOH Unemployment, Job Insecurity and Health (UJIH) Scientific Committee using a cross-sectional study design. A researcher-designed online survey questionnaire was deployed using the Google form platform. The questionnaire was shared with OH professionals through the ICOH community [16]. The questionnaire (see Appendix A) consisted of open-ended and closed questions in English. It was divided into the following four sections: respondents’ views on their government and employer responses to COVID-19, work-related access to personal protective equipment (PPE), the impact of COVID-19 on work and employment (especially in relation to vulnerable groups), and demographic data of respondents. This questionnaire was shared via email within the ICOH community through ICOH chairs and secretaries of all the ICOH scientific committees and all ICOH country secretaries. The inclusion criteria were that the respondents were members of ICOH. There were no exclusion criteria. However, in practice, the respondents were most likely to be those contacted directly by e-mail (that is, Chairs and Secretaries as listed above). Data was collected over a period of two weeks in May 2020. Given the short window of data collection, there was limited opportunity for other members to respond. Therefore, although the total ICOH membership is just over 2000, the denominator is likely to be smaller than this. Participation in the survey was voluntary and anonymous.

This paper focuses specifically on the respondents’ perceptions of the impact of the COVID-19 pandemic on vulnerable groups. The relevant questions to this study were: which groups of workers might be further disadvantaged by the pandemic, whether their governments could take mitigating actions and if so, what actions (Q21, 22, and 23). The quantitative data were analyzed using IBM Scientific Package for the Social Sciences (SPSS version 25, IBM Corporate Headquarters, 1 New Orchard Road, Armonk, NY 10504-1722, USA). The analyzed data are presented using simple descriptive statistics, such as frequency tables and percentages.

## 3. Results

### 3.1. Sociodemographic Characteristics of Study Participants

By the end of the two-week survey period, 165 responses from 52 countries were received. The responses were divided into continents, which would allow for some further analysis. Briefly, most of the respondents were from Europe (42.4%), followed by Africa (30.9%), and Asia (15.2%). The highest reported professional group was occupational health physicians (39.4%), followed by occupational health researchers (26.1%), and academics (21.8%). The sociodemographic characteristics of the respondents are summarized in Table 1 (previously published by the authors in an ICOH newsletter [16]).

In this paper, the responses relating to questions about vulnerable workers (Q21–23) are reported. The full list of the thirty questions is outlined in Appendix A. The perceived disadvantage by group on account of COVID-19 is tabulated by continent in Table 2.

Globally, all the respondents believed that at least one group of workers (chosen as possible vulnerable groups) would be further disadvantaged as a result of COVID-19. This survey question allowed more than one group to be selected. Of the groups listed, most responders indicated that those in precarious employment (126, 79.2%) would be further disadvantaged, followed by those in the informal sector (109, 68.6%), migrant workers (103, 64.8%), the unemployed (100, 62.9%), those with a disability or long-term health condition (97, 61%), and older workers (89, 56%). We did not specify any age for “older workers” because we felt this could vary from country to country, so we left it to respondents’ local interpretations of who were considered “older workers” in their contexts. Few respondents (33, 20.8%) felt that women would be further disadvantaged. Only a very few responders (9, 5.7%) suggested any other groups of workers that might suffer further detriment due to COVID-19 (for example, one mentioning young workers and one suggesting the tourism sector).

### 3.2. Continental Patterns

If we look at the results by continent, similar patterns emerge but some variations are also evident. We concentrate on the responses from Africa, Asia, and Europe because the numbers of respondents from America and Australia were too small. The responses from Africa reflected the global pattern, with the top two categories being those in precarious employment and those in the informal sector (86% for both), followed by the unemployed (72%). The top three categories for Europe were those in precarious employment (83%), migrant workers (66%), and the unemployed (65%). The pattern from Asia was the most different to the averaged global one, as the predominant group for our respondents from Asia were migrant workers (73%) followed by those in precarious employment and those with disabilities (both 62%).

The responses to whether their governments could take action to mitigate the impact of this pandemic on these groups of vulnerable workers is tabulated by continent in Table 3.

Overall, the majority (67%) believed that their government could take some actions that could mitigate further disadvantage to the more vulnerable groups of workers. Again, if we concentrate on the three continents with the largest number of responses, namely Africa, Asia, and Europe, we find that the responders from Asia (89%) were the most likely to indicate that their governments could take some actions to mitigate against further disadvantages. Responders from Africa and Europe had similar proportions (62%) believing that their governments could take such mitigating actions.

### 3.3. Mitigating Actions by Governments

The qualitative responses to Q23, which reflect suggestions of the actions that governments could take, are listed in Appendix B. We could consider the suggestions in the following four categories (which are not mutually exclusive): (i) General actions, that is, actions that would benefit most of the population and not necessarily limited to those who are more vulnerable. Examples include improving control of the pandemic or opening up the economy with appropriate risk assessments and controls in place. Whereas such measures would likely improve the situation for most workers, these were not specifically targeted at the more disadvantaged populations. (ii) Specific action for a group, for example, returning migrants safely to their hometowns. (iii) Focused support, for example, improving the social and economic support for vulnerable groups, including unemployment benefits (for the unemployed) and other financial assistance, food delivery, and providing a social safety net. Retraining vulnerable workers to have transferable skills or IT skills was also suggested. (iv) In-depth review of underlying inequalities, i.e., there were also calls for more fundamental reviews and actions to address the systemic inequalities and discrimination that underpin why such groups are more vulnerable to a pandemic.

## 4. Discussion

Occupational health has recently been described as “the thin line protecting the front line” in this COVID-19 pandemic [17]. This was in the context of OH services to healthcare workers in the UK. Indeed, a significant number of papers in the OH and COVID-19 literature have pertained to frontline healthcare staff [18,19,20,21]. However, this description is likely to be apt for OH professionals in most sectors globally as they are involved in assessing, advising, and monitoring those working and aiming to return to work in the midst of this pandemic. More recent papers related to COVID-19 and work, mainly from an OH perspective, address subjects as diverse as the reporting of occupationally acquired COVID-19 in all sectors [22] and the safe return to work of those who had been “shielding” because of COVID-19 due to possible risk factors, and of those who had contracted COVID-19 [23]. Therefore, OH professionals are well placed to have informed and cogent opinions on which groups of workers might be or become more adversely affected by this pandemic. Therefore, although our data consists of subjective responses to our questions, we believe that the opinions of OH professionals are especially valuable in this context. To our knowledge, there has, so far, not been previously published work on COVID-19 and vulnerable workers from the perspective of OH professionals.

Globally, our responders felt that those who were either in less secure jobs, that is, in precarious employment (including low paid, “zero-hour” contracts) (79%) and informal work (such as street food vendors and waste pickers) (69%), or who were unemployed (63%), were the most at risk of further disadvantage from this pandemic. This correlates well with other available literature. For example, the International Labor Organization (ILO) describe the 1.6 billion informal economy workers as being amongst the most vulnerable in the labor market. ILO estimated a decline in their earnings of 60% globally in the first month of the pandemic, with the largest regional declines expected to be 81% in Africa and Latin America [24]. In addition, job insecurity and unemployment are already known to be strongly associated with poorer health. Indeed, both are said to cause ill health [25]. For example, those in insecure work are 1.25 more likely to suffer ill health and 2.5 more likely to suffer mental ill health than those in secure jobs [26].

The next group thought to be at risk of further disadvantage were migrant workers (65%). Migrants tend to work in sectors that put them at greater risk of COVID-19 infection (such as farms and food processing facilities) [3], but also typically have temporary or informal work arrangements, with low wages and social protection, leaving them more vulnerable to the economic consequences of this pandemic [27] and requiring more co-ordinated responses from government agencies [28].

Those with disabilities or long-term health problems (61%) and older workers (56%) were the next two groups that our respondents felt would fare worse in this pandemic. Even pre-COVID-19, it was noted that disabled employees in the UK were more likely to be in part-time jobs, to be paid less, and to have their employment rights infringed despite legislation intended to prevent this [29]. It has been argued that those with disabilities are particularly socioeconomically disadvantaged (for example, that they are more likely to lose their jobs) [30] and have had their rights further eroded [3] as a result of the COVID-19 pandemic. Similarly, older persons can face worsening of their socioeconomic situations, especially those who are poorer and socially excluded. Previous pandemics (such as MERS) led to higher unemployment and underemployment in older workers as compared with younger workers. In addition, it is reported that COVID-19 is escalating age discrimination and stigmatization [31].

Only 21% of respondents thought that women workers would suffer further disadvantage from the pandemic. This is maybe surprising as women are overrepresented in lower paid and unprotected jobs [26]. Moreover, the economic downturn in the service sector is said to be a further contributor to the disproportionate effects of COVID-19 on women workers [32]. It is unlikely that the demographics of our respondents (52.7% male to 46.7% female) would explain this finding. One possible explanation could be that women workers are not traditionally listed as one of the groups of “vulnerable workers” (such as in [4]). However, current evidence [26,32] suggests that OH professionals should be more cognizant of this group’s particular vulnerability to the socioeconomic effects of this pandemic.

When we look at the continental pattern of responses to Q22, although we note many similarities between the continents, we also see some interesting differences. Precarious work features at or near the top for most continents (Africa 86%, Asia 62%, and Europe 83%). In contrast, those working in the informal sector were more likely to be considered to be a vulnerable group in Africa (86%) than in Europe (60%) or Asia (58%). This may reflect how those in this sector are organized. For example, it is possible that more workers are in this sector in Africa than in Europe. However, this may not fully explain the difference in perception of vulnerability, given that there are likely to be many in this sector in Asia and they were not rated there as being a more disadvantaged vulnerable a group as compared with migrants (73%) or those with disabilities (62%). Indeed, in Asia, migrants (73%) were the most likely to be considered further disadvantaged by COVID-19. This may reflect the nature of workforces on the Asian continent, as well as there being little or no structural support for this group of workers. Women (8%) and the unemployed (42%) were the two groups the least likely to be rated to be further disadvantaged in Asia. As noted in the global section of our analysis, women were generally not thought to be further disadvantaged, and this was reflected in the continental figures, i.e., Europe 29% and Africa 8%. As we commented above, this does not reflect concerns in the current literature that women are a particularly vulnerable group in these COVID times and may be a learning point for OH professionals. Responses related to disability were consistent across the continents, i.e., Africa 62%, Asia 62% and Europe 60%. The responses related to older workers were similar from Africa (58%) and Europe (60%) but were lower from Asia (42%).

In reply to Q22, globally, the majority (67%) believed that their government could take actions to mitigate the effects of the pandemic in these already disadvantaged groups. Amongst the three continents with the most responses, those from Africa and Europe were similar (62%) and the highest number believing that their governments could take mitigating measure were from Asia (89%). Taken in isolation, Q22 could be ambiguous, as it would not be clear, for example, whether respondents thought that their governments could take further actions because not enough had been done so far, or whether the respondents thought their governments had already taken action but there was more that could be done. However, the real value of Q22 was to elicit the qualitative responses to Q23. We wanted to see what our respondents might suggest in terms of actions their governments could take in this context.

In response to Q23, we were particularly interested by those suggestions of fundamental reviews of inequalities and discrimination that lead to some populations being more disadvantaged and leading to their greater susceptibility to pandemics. This resonates well with other published papers that have examined this aspect of the pandemic. For example, we note that inequities in terms of race and ethnicity [33] and other societal inequalities [3] lead to worse health outcomes in this COVID pandemic. The multidimensional nature of disadvantage in this context, which includes poverty, poorer health and education, less secure work or unemployment, multifaceted discrimination, and lack of social support, leads to various vulnerable populations suffering much worse effects from the pandemic in terms of both their health and their ability to work [11]. In turn, economic loss can lead to poorer health (for example by not being able to afford adequate nutrition) and poorer health can lead to a decreased ability to work. These vicious cycles between work and ill health are especially acute in disadvantaged populations and made even be worsened by the health and socioeconomic impacts of this pandemic. We believe, therefore, that enlightened approaches to recovery from this pandemic should not merely aim for a “return to normal”, but rather, seek to ameliorate the situation for disadvantaged populations in a lasting way. For example, a United Nations Development Programme (UNPD) report [34] on recovery from the COVID crisis noted that responses should be multidimensional, be viewed through an equity lens, and focus on people’s long-term capabilities. The UNDP uses the capability approach [35], which is an economic theory that places as its central focus the individual’s capabilities (what an individual is able to be and do) and “functionings” (this is a term used in the capability approach and means “what the individual is able to achieve in terms of her capabilities”). This UNDP report also highlighted the plight of those in precarious employment in this pandemic, such that government responses must “reach those weak links of the social and economic fabric as well as those who have already been left behind, supporting their basic capabilities and enabling subsistence” [34]. It encourages policies enabling social protection in ways that that reduce existing inequalities and that empower individuals to achieve their development. In the capability literature, health is seen as a prerequisite to achieving one’s other capabilities [36] and the ability to live a life that one values [37], and therefore maintaining and protecting the health of vulnerable populations is especially important in the midst of a pandemic. One should also note the importance of addressing disadvantage in ways that respect the individuals concerned [26]. Indeed, it has been argued that this current pandemic has offered us the opportunity to learn to do this [38].

This study has some strengths and weaknesses. On the positive side, it was a cross-sectional survey done at the height of the pandemic, therefore, it provided us with time-sensitive information from a relevant period (May 2020) during its unfolding of the pandemic. It also captured data from different countries, which allowed us to make regional comparisons (by continent). However, we do not have a denominator, that is, the number of possible respondents who could have completed the survey. Nevertheless, authors of a survey in a comparable context argued that the lack of denominator did not impact on their outcome [39] and we agree with them. We do not believe that this affects our findings either. Another criticism that could be levelled at our survey is that we are reporting subjective opinions. We have argued above that OH professionals are uniquely placed at the interface of work and health. Their active involvement in this pandemic [17], therefore, provides us with valuable insights from arguably the group most conversant with our survey questions. We also note that we had not made it explicit in Q21 whether we meant vulnerability to further health or socioeconomic deterioration. It is the latter deterioration that was the focus of our interest. It is possibly fortunate that our colleagues interpreted the question as we meant it, as we can infer by their responses in Q23, which all describe socioeconomic solutions or strategies. Nonetheless, if this survey were repeated in the future to assess whether OH professionals’ views had changed in time, it would be preferable to specify “socioeconomically disadvantage” in Q21. A future study might also clarify Q22 further by having different parts to this question, for example, by asking respondents first whether they thought their governments were already taking relevant mitigating actions. Nonetheless, in our study, we were more interested in using Q22 as a preamble to the qualitative free-text replies to Q23, so we do not feel that the current wording of Q22 had a detrimental impact on our study findings. However, the methodology used, which was a bespoke questionnaire (as there is no validated questionnaire that would have elicited the information of interest) and the low numbers in our study, limit the generalizability of our findings.

## 5. Conclusions

In conclusion, our respondents most often identified workers with job insecurity (that is, those in precarious employment or in the informal sector) as the disadvantaged groups likely to suffer further detriment due to COVID-19. The majority felt that their governments could take some action to mitigate these effects. There were suggestions of short-term alleviation such as financial and social support. There were also calls for more fundamental reviews of the underlying inequalities that leave populations vulnerable to a crisis such as COVID-19. These approaches resonate with the recommendations of a UNPD report [34]. Occupational health professionals are well placed to promote the importance of equity in future strategies and policies in the changing world of work [40]. Indeed, in relation to vulnerable workers, it has been argued that OH professionals have a key role [4] and a moral duty [6] to encourage policies that improve social justice and reduce inequalities. We also hope that by highlighting the plight of workers in insecure work during this pandemic, this paper will encourage future research, maybe around the systemic inequalities that lead to this situation. Fairer societies will likely be more resilient to future global crises.

## Figures and Tables

**Table 1:** Sociodemographic characteristics of respondents.

Variable	Category	Distribution N (%)
Gender	Female	77 (46.7)
	Male	87 (52.7)
	Do not wish to say	1 (0.6)
Age	21–30	11(6.7)
	31–40	33 (20.0)
	41–50	41 (24.8)
	51–60	40 (24.2)
	61–70	36 (21.8)
	71–80	3 (1.8)
	>80	1 (0.6)
Profession	Academics	36 (21.8)
	Occupational health physicians	65 (39.4)
	Occupational hygienist	27 (16.4)
	Occupational health researchers	43 (26.1)
	Others	20 (12.1)
Continent	Africa	51 (30.9)
	America	12 (7.3)
	Asia	25 (15.2)
	Australia	5 (3.0)
	Europe	70 (42.4)

**Table 2:** Perceived disadvantage by group on account of COVID-19.

Responses by Continent/Numbers	1. Disability (%)	2. Migrants (%)	3. Women (%)	4. Unemployed (%)	5. Precarious (%)	6. Informal (%)	7. Older (%)	8. Others (%)
Africa/50	31/50 (62)	29/50 (58)	8/50 (16)	36/50 (72)	43/50 (86)	43/50 (86)	29/50 (58)	3/50 (6)
America */12 (North 5, South America 7)	7/12 (58.3)	8/12 (66.7)	2/12 (16.7)	10/12 (83.3)	9/12 (75.0)	10/12 (83.3)	9/12 (75.0)	2/12 (16.7)
Asia/26	16/26 (61.5)	19/26 (73.1)	2/26 (7.7)	11/26 (42.3)	16/26 (61.5)	15/26 (57.7)	11/26 (42.3)	0 (0)
Europe/65	39/65 (60)	43/65 (66.2)	19/65 (29.2)	42/65 (64.6)	54/65 (83.1)	39/65 (60)	37/65 (56.9)	4/65 (6.2)
Australia */5	4/5 (80)	4/5 (80)	2/5 (40)	1/5 (20)	4/5 (80)	2/5 (40)	3/5 (60)	0 (0)
Not specified/7								
Total/165	97/165 (61)	103/165 (64.8)	33/165 (20.8)	100/165 (62.9)	126/165 (79.2)	109/165 (68.6)	89/165 (56)	9/165 (5.7)

* not included in analysis due to small numbers 1, those with a disability or long-term health condition; 2, migrant workers; 3, women; 4, unemployed; 5, those in precarious employment (including low paid, “zero-hour” contracts); 6, informal workers such as street food vendors, waste pickers, etc.; 7, older workers; 8. others.

**Table 3:** Perceptions of the action by government to mitigate the impact of COVID-19.

Continent	Participants	Yes	Percent
Africa	50	31	62
America	12	12	100
Asia	26	23	88.5
Europe	65	40	61.5
Australia	5	4	80
Not specified	7		
Total	165	110	66.7

## Data Availability

The data presented in this study are available on request from the corresponding author. The data are not publicly available in order to maintain confidentiality of study participants and only aggregated data will be available.

## Appendix A. Full List of Questions in Survey Questionnaire

**Section 1: Background to COVID-19**	
***Q1: In your opinion what is the quality of the COVID-19 information provided by the Government of your country?**	
*Excellent	
*Good	
*Average	
*Poor	
***Q2: In your opinion what is the quality of the COVID-19 response provided by the Government of your country?**	
*Excellent	
*Good	
*Average	
*Poor	
**Q3: In your opinion what is the quality of the COVID-19 information provided by your Employer?**	
*Excellent	
*Good	
*Average	
*Poor	
**Q4: In your opinion what is the quality of the COVID-19 response provided by your Employer?**	
*Excellent	
*Good	
*Average	
*Poor	
**Q5: What strategies have been implemented as of 1st May 2020 with regards to the response to COVID-19 by your Government ? (choose all that apply)**	
*Social distancing without lockdown	
*Local/partial lockdown (geographical boundaries)	
*Local/partial lockdown for ‘at risk groups’	
*National lockdown	
*Other: Please comment	
**Q6: What media platforms have you used to access information on COVID-19? (choose all that apply)**	
*whatsapp	
*facebook	
*twitter	
*Instagram	
*LinkedIn	
*National news channel	
*Official Government Committee Announcement	
*National Health and Safety Body website	
*Websites of other organisations, e.g., WHO, ILO, CDC, NIOSH, HSE, etc.	
*Popular celebrity or leader	
*Other: Please comment	
*None	
**Q7: What is your current reaction to the COVID-19 situation as of 1st May 2020? (choose all that apply)**	
*Shock	
*Denial	
*Frustration	
*Depression	
*Experiment	
*Decision	
*Integration	
*Other: Please comment	
**Section 2: Availability of Personal Protective Equipment (PPE) for health workers**	
**Q8: Do you think your country has provided adequate PPE for health workers likely to come into contact with COVID-19 cases?**	
*Yes	
*No	
*Don’t know	
**Q9: Is there any discrimination in the supply of PPE?**	
*Yes	
*No	
*Don’t know	
**Q10: In case you have answered yes in Q9, please may you comment on instances of discrimination and an other factors leading to poor supply of PPE?**	
Please comment:	
**Q11. Which group of persons have better access to PPE in your country? (choose all that apply)**	
*Police	
*Security personnel	
*Politicians	
*Health workers	
*Delivery staff	
*Private sector workers having better access than the public sector workers	
*Males getting preferential access to PPE	
*Females getting preferential access to PPE	
*Other: Please comment	
**Q12: Have you come across situations in your country where PPE is not readily available for health workers?**	
*Yes	
*No	
*Don’t know	
**Q13: Can you comment and provide examples of poor PPE access for health workers?**	
Please comment	
**Q14. What type of PPE is NOT readily available to health workers as of 1st May 2020 in your country? (choose all that apply)**	
*Surgical mask	
*N95 mask	
*FFP3 respirator	
*Face shield	
*Hazmat suit	
*Other: Please give examples	
**Q15: What innovative schemes have been developed in your country for PPE (face mask) and Sanitisation products production?**	
*Local production of face masks	
*3D printing schemes	
*Making alcohol gel from byproduct of the brewing industry	
*Other: Please give examples	
**Section 3: Impact of COVID-19 on work/employment**	
**Q16: What kind of measures are in place in your country to cushion the effect of COVID-19 on work and employment? (choose all that apply)**	
*Protection of workers in the workplace (e.g., strengthening OSH measures, preventing discrimination)	
*Supporting employment and income (e.g., social/unemployment benefits)	
*Compensation system for occupationally acquired COVID-19	
*Others, specify	
*None	
**Q17: Do you consider the measures in the question above (Q16) to be adequate?**	
*Yes	
*No	
**Q18: In case you have answered No in Q17, please may you be able to comment below?**	

**Q19: What other measures do you think would help cushion the job security effects of COVID-19? Please may you comment below**	

**Q20: What do you feel the future holds post COVID-19 for work and employment?**	
*No hope	
*Some hope	
*Hopeful	
*Good future	
*Other: Please explain	
**Q21. Which groups of workers, if any, do you feel may become even more disadvantaged as a result of COVID-19? (choose all that apply)**	
*None	
*Those with a disability or long term health condition	
*Migrant workers	
*Women	
*Unemployed	
*Those in precarious employment (including low paid, zero hour contracts)	
*Informal workers such as street food vendors, waste pickers, etc.	
*Older workers	
*Other: please explain	
**Q22. Do you feel there are any actions your Government could take to mitigate the effects on any groups in Q21 above?**	
*Yes	
*No	
**Q23: In case you have answered Yes in Q22 above, please may you be able to comment below:**	

**Section 4: About you**	
**Q24. What country are you based in?**	

**Q25. What city are you based in?**	

**Q26: What is your gender?**	
*Male	
*Female	
*Other	
**Q27: What is your age?**	
*21–30	
*31–40	
*41–50	
*51–60	
*61–70	
*71–80	
>80	
**Q28: What is your profession?**	
*Academic	
Occupational health physician	
*Occupational health nurse	
*Occupational health researcher	
*Occupational hygienist/industrial hygienist	
*Other: Please specify	
**Q29: What type of organisation do you work for? (choose all that apply)**	
*Public sector	
*Private sector	
*Agriculture and other rural sectors	
*Basic metal production	
*Chemical industries	
*Commerce	
*Construction	
*Education	
*Financial and professional services	
*Food, beverage, tobacco	
*Forestry, wood, pulp and paper	
*Health services	
*Hotels, tourism, catering	
*Mechanical and electrical engineering, electronics and IT	
*Media, cultural, graphics	
*Mining	
*Oil and gas (production and refining)	
*Postal and telecommunication services	
*Shipping, ports, fisheries	
*Textiles, clothing, footwear	
*Transport (aviation, rail, and road)	
*Utilities (water, gas, electricity)	
*Other, please specify:	
**Q30: In case you have any further comments on further questions or areas of improvement for this survey, would be most appreciated. Please comment below:**	

## Appendix B. Answers to Q23

Q23 In case you have answered Yes in Q22 above, please may you be able to comment below.
Answers:
**Administration/General Policy**
Make sure to review statistics of employment among these groups.
It is necessary to analyse, identify, and use the opportunities for people.
Guidelines.
Review labour laws to accommodate the pandemic.
More supportive and inclusive policies, universal basic income.
Develop mitigating plans for this.
Law enforcement.
Subventions (but not enough controlled in practice, especially among private sectors).
Introduce permanent infrastructure to deal with poverty and inequality.
I think society as a whole will need to re-configure to meet demands, retraining, new types of job, new ways of working, e.g., how to continue education, health, childcare, transports, essential services in a COVID world The government, national bodies might as well start considering what this new, long-term COVID world is going to look like now.
Do something about discrimination against specific racial groups
To be honest, I am not sure what actions may be taken. However, I feel that these actions will need to be innovative and feasible in the long term.
Extend the intended duration of various support programs and broaden access to those not receiving benefits now (some casual workers, temporary visa holders, international students).
Teach business people how to spot possible corruption trends and how to prevent it.
Plan a relief fund.
Improvement on wages.
Mass testing.
Social and economic relief.
Tax the rich and give money to the poor.
Stop political appointees as advisers.
**Economic Sector/Industry**
Incentive industrial production.
Open up certain industries and do away with ridiculous regulations (no alcohol/cigarette sales, sale of closed shoes allowed, but not open, no T shirt sales, but Golf shirts allowed etc!!!
Permit business with strict enforcement of usage of masks, sanitizers, and social distancing.
Allowing people to start working (e.g., vendors) but after solid training on H&S and under strict rules.
Re-opening the businesses ASAP.
Haul the economy and give monthly stipends.
Same as Q19, it should not be compartmentalised action, instead it should be global action that will stimulate the economy.
Retraining of employees to be equipped with transferable skills and IT skills to be ready for an uncertain world. Facilitation of IT adoption by companies to enable new ways of working or new business model.
Keep the healthy older worker with experience for coaching and mentoring purposes,
policy to relax debt payments.
Economic reforms.
Activate economy responsibly, reduce public employment and spending where it is not needed.
Our government steps to boost for the economy are very welcome for all the Indians.
Redraft economic policy to favour local corporations.
Do away with their racist policies and ensure that they form business compacts with big business and high net worth individuals to release cash reserves into the economy.
Social and economic relief.
Government taking steps to review the economic impacts.
Economic support month.
Same as Q19, it should not be compartmentalised action, instead it should be global action that will stimulate the economy.
Support employers.
Training people how they can become entrepreneurs and how to manage their small business successful. Provide finances to start small business and do audits to see are they managing and providing job opportunities for others. Have audits done and give feedbacks on trends.
Identify those at risk and clear guidance on RTW and safe work practices and social welfare contributions if not possible to RTW.
Job retraining programs, equity in access to financial support.
Loans and grants.
Prefer employment in essential services area, be local employ local and be state employ state.
**Social Support**
Strengthening social support to disadvantaged workers.
Provide more support to families with children to protect women’s employment, set up social support and empower welfare system to support vulnerable and fragile workers social safety net support; re-training workers who are at high risk to do other work; sick pay for all workers to reduce spread of virus.
This is now being done, i.e., people over 65 years old and with chronic diseases (diabetes, oncology, serious problems with the cardiovascular and bronchopulmonary systems) are completely relieved of work in state institutions with a fully preserved salary. In private institutions, everything is more complicated.
Social protection.
Social security schemes by Social Security Organization.
Social Protection and incomes Public Health Services.
A real compensation programs.
Unemployment benefits and compensation.
Introduce unemployment allowance, introduce social welfare policies.
Food delivery to unemployed families.
Grants and other benefits to be provided on need not race or friendship or political affiliation.
Direct future financial supports to those groups.
Financial help would have helped them.
Direct transfer of financial benefits to the workers and ensuring safe transport to migrant workers home town.
The government should prepare financial assistance, paying particular attention to older and disabled workers, who are in difficult situation and have bigger needs.
Financial support, development projects.
Financial support.
Improve financial resources for those in need.
Expedite financial assistance initiated by government and enforce lockdown measures equally all over the country
help targeted groups and finance costs from the rich and wealthy (companies).
Some income to lowest strata of workers.
**Migrant Workers**
Strong support for migrant workers.
Direct transfer of financial benefits to the workers and ensuring safe transport to migrant workers home town.
The biggest problem is with migrants, but there are also great successes in this environment (isolation, increased medical control, social benefits).
**Others**
Government is helping all employees to sustain.
Domestic violence has increased due to couple having to be locked down and home isolate and home school children. This has been very difficult particularly on working women.
Provide resources to manage long term health conditions (co-morbidities).
Awarding of tenders to appropriately qualified service providers for cleaning services. Currently there is corruption and government has not tackled the issue appropriately.
The German government is considering all/many of these aspects, but finite outcomes are not all certain yet.
We are all exposed in one way or another until certain factors are determined that make the care, diagnosis, and treatment of said pathology.
Age is not the criteria. Physical fitness and mental ability.
